# Spatiotemporal information transfer pattern differences in motor selection

**DOI:** 10.1186/1471-2202-12-S1-P261

**Published:** 2011-07-18

**Authors:** Joseph T Lizier, Jakob Heinzle, Chun S Soon, John-Dylan Haynes, Mikhail Prokopenko

**Affiliations:** 1 Max Planck Institute for Mathematics in the Sciences, 04103 Leipzig, Germany; 2 CSIRO Information and Communications Technology Centre, Marsfield, NSW 2122, Australia; 3 School of Information Technologies, The University of Sydney, NSW 2006, Australia; 4 Bernstein Center for Computational Neuroscience, Charité–Universitätsmedizin Berlin, 10115 Berlin, Germany; 5 Max Planck Institute for Human Cognitive and Brain Sciences, 04103 Leipzig, Germany; 6 Duke-NUS Graduate Medical School, Singapore, Singapore; 7 Graduate School of Mind and Brain, Humboldt Universität zu Berlin, 10099 Berlin, Germany

## 

Analysis of information transfer between variables in brain images is currently a popular topic, e.g. [1]. Such work typically focuses on *average* information transfer (i.e. *transfer entropy*[*2*]), yet the *dynamics* of transfer from a source to a destination can also be quantified at *individual* time points using the *local transfer entropy* (*TE*) [3]. This local perspective is known to reveal dynamical structure that the average cannot. We present a method to quantify *local TE* values in time between source and destination *regions* of variables in brain-imaging data, combining:

a. computation of inter-regional transfer between two regions of variables (e.g. voxels) [1], with

b. the local perspective of the dynamics of such transfer in time [3].

Transfer is computed over samples from all variables – there is no training in or subset selection of variables to use.

We apply this method to a set of fMRI measurements where we could expect to see differences in local information transfer between two conditions *at specific time steps*. The fMRI data set analyzed (from [4]) contains brain activity recorded from 7 localized regions while 12 subjects (who gave informed written consent) were asked to freely decide whether to push one of two buttons (with left or right index finger), whenever they felt the urge to do so, and to press the button immediately on deciding . To our knowledge, this is the first analysis of transfer entropy on a local temporal scale in brain-imaging data (at specific time points rather than via sliding windows).

**Figure 1:**
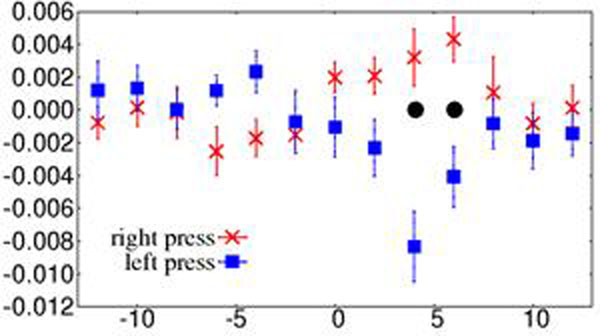
Difference in TE(pre-SMA → left motor) and TE(pre-SMA → right motor) versus time after button press for subject 1. Error bars are std. err. over presses. Significant difference between left and right button press indicated by · at t=4 and 6 sec (no aggregation over consecutive time steps).

Significant differences in the local TE between left and right button presses are revealed in a significant number of subjects (7) by:

a. examining the *difference* in local TE from a single source region (e.g. pre-SMA) into left and right motor cortex respectively (e.g. see Figure 1 for subject 1); and

b. *aggregating* local TE differences across 2 to 3 consecutive time steps (e.g. t=2,4 and 6 sec. after button press).

Additionally, thresholding of these TE differences can decode the button push with a (statistically significant) mean of 65% accuracy across subjects. These measurements of local TE correlate well with the role of these regions in executing the motor response here [4]. We confirm that local TE can be used to reveal differences in task-based dynamical information transfer, with potential for the technique to be improved in the future.
